# Analgesic effect of simultaneously targeting multiple pain processing brain circuits in an aged humanized mouse model of chronic pain by transcranial focused ultrasound

**DOI:** 10.1063/5.0236108

**Published:** 2025-02-19

**Authors:** Min Gon Kim, Chih-Yu Yeh, Kai Yu, Zherui Li, Kalpna Gupta, Bin He

**Affiliations:** 1 Department of Biomedical Engineering, Carnegie Mellon University, Pittsburgh, Pennsylvania 15213, USA; 2 Department of Medicine, University of California at Irvine, Irvine, California 92697, USA; 3 Neuroscience Institute, Carnegie Mellon University, Pittsburgh, Pennsylvania 15213, USA

## Abstract

Low-intensity transcranial focused ultrasound (tFUS) has recently been shown to noninvasively and non-pharmacologically modulate pain hypersensitivity with high spatial specificity and deep brain penetration. However, the lack of knowledge about its effectiveness for pain management in older subjects vulnerable to severe pain who are also at increased risk of cognitive impairment, presents significant challenges. Additionally, current opioid pain treatments require hospital visits, limiting unwanted serious side effects with multiple liabilities, and device-based pain treatments are typically administered at medical facilities with bulky and expensive equipment, limiting accessibility and thus highlighting the need for at-home non-pharmacological treatment options. Here, we present a more accessible, noninvasive tFUS pain treatment strategy for senior subjects. This approach involves simultaneously targeting multiple pain-processing circuits using a battery-powered, compact, and low-cost ultrasound analog front end (UAFE). We developed and evaluated the performance of the UAFE capable of generating sufficiently high-amplitude output with significantly lower noise levels compared to a commercial transmitter. Using a humanized sickle mouse model of chronic hyperalgesia, we found that tFUS stimulation targeting multiple pain-processing circuits effectively reduces heat hyperalgesia in aged female mice. In addition to its efficacy, our behavioral-based safety assessment revealed no adverse effects on motor functions. These results suggest that using a battery-powered, compact UAFE to simultaneously target multiple pain-processing circuits can effectively suppress heat pain-related behaviors in aged female sickle mice without negatively impacting motor coordination and balance. This highlights the potential for further development of fully home-based tFUS pain treatment for seniors.

## INTRODUCTION

Treating pain in the senior population presents several challenges.^1^ Alterations in neuroanatomy, physiology, and biochemistry of nociceptive pathways can affect pain thresholds and tolerance, leading to severe pain and an increased risk of cognitive impairments,^2,3^ which subsequently impacts the overall quality of life.^4^ With increasing age, mobility and access to care become significantly impacted, necessitating that therapies be administered at home. Therefore, developing an effective and safe pain treatment option for the elderly is an urgent unmet need.^5^ While pharmacological strategies have been used for pain management,^6^ prior studies have demonstrated analgesic effects in the general population but not necessarily in the elderly.^7^ Given that older adults often experience severe pain or inadequate relief from opioid doses equivalent to those used in younger adults, higher and more frequent dosing of opioids may be required. This, in turn, increases the risk of dose-related side effects and fear of addition.^8^ Consequently, clinical practice guidelines emphasize the need for developing new non-pharmacologic and noninvasive pain interventions.^9^ Noninvasive brain stimulation techniques such as transcranial magnetic stimulation (TMS) and transcranial direct current stimulation (tDCS) have shown merits for treating pain and depression in both younger and older adults by identifying optimal treatment paradigms.^10,11^ However, TMS and tDCS have limitations including their broad targeting of brain areas rather than specific regions and restricted penetration depth.^12,13^ In contrast, low-intensity transcranial focused ultrasound (tFUS) offers high specificity and deep penetration depth, demonstrating promising experimental evidence that tFUS noninvasively elicits or inhibits brain activity by varying tFUS parameters^14–17^ and treats a myriad of neurological disorders including epilepsy,^18^ depression,^19^ and essential tremor.^20^ Inspired by these promising results, researchers have extended the use of noninvasive tFUS stimulation with a high degree of specificity and penetration depth to demonstrating a significant potential for pain treatment as evidenced by studies in healthy adults, chronic pain patients, and small animals.^21–25^ However, these studies for developing pain treatment have predominantly focused on middle-age adults subjects, and rodents,^21–25^ with limited research involving chronic pain in the elderly.^24,25^ This gap in research makes it challenging to assess the utility of tFUS stimulation in older populations. Therefore, further studies are needed to explore the potential and examine the capability of tFUS for managing pain in the elderly. Additionally, inadequate pain management in older adults often faces significant barriers such as limited mobility, multiple comorbidities, and the challenges of traveling to clinics that use bulky and expensive device-based approaches.^26^ To address these challenges and improve accessibility, there is also a strong need for easy-to-use, compact, and low-cost home-based tFUS treatments.

In this study, we develop a battery-powered, compact, and low-cost ultrasound analog front end (UAFE) and demonstrate significant amelioration of pain-associated behaviors in older female rodents, considering the unique challenges of pain treatment in older female adults who have been reported to experience more intense pain than males.^27,28^ We hypothesize that simultaneously targeting multiple pain processing brain circuits could effectively suppress heat hyperalgesia in older female humanized mouse model of sickle cell disease (HbSS-BERK), over 99% human sickle hemoglobin expression, and exhibit severe acute and chronic pain behaviors, which increase with age.^29,30^ We designed, simulated, developed, and evaluated the performance of a custom-built UAFE, comparing its effectiveness with that of a commercial transmitter. We then investigated the impact of age on response to painful heat stimuli by comparing 8-to-9-month-old and 18-to-19-month-old female BERK mice. We evaluated the efficacy of tFUS stimulation targeting multiple pain processing circuits including the hindlimb of the primary somatosensory cortex (S1HL) and agranular insular (AI) by comparing pre-stimulation baselines with multiple stimulation control groups. Blind assessments were conducted to rigorously evaluate the unilateral effects of tFUS on specific brain circuits in the left hemisphere. Additionally, we assessed whether tFUS stimulation to multiple pain processing circuits adversely affected motor function through a behavioral-based safety study. Our findings demonstrate that tFUS device developed by us effectively suppresses hyperalgesia and holds potential for home-based use in elderly individuals experiencing severe pain.

## RESULTS

### Design and simulation of the custom-built ultrasound analog front end (UAFE)

**FIG. 1.**
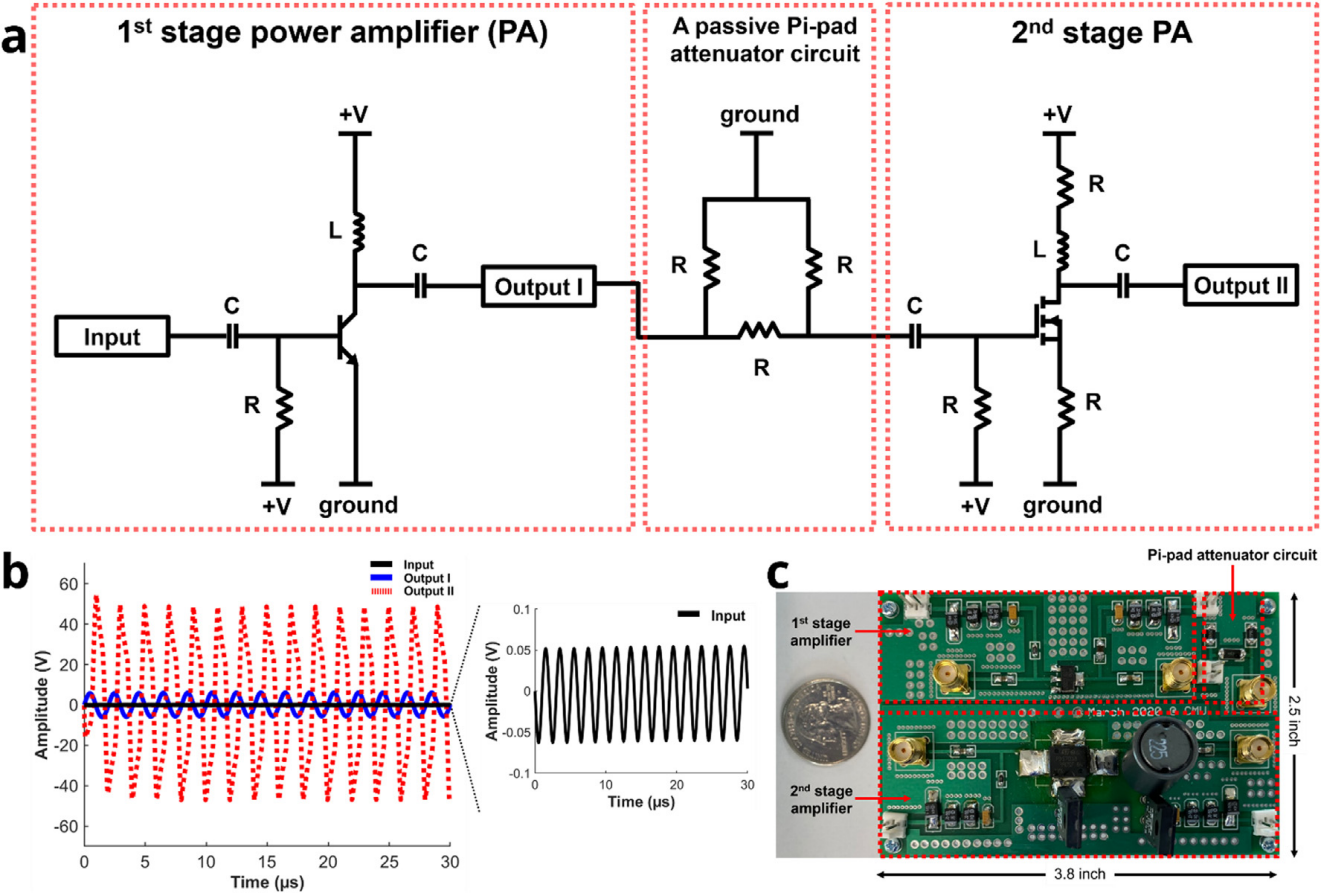
Circuit schematic and performance of the custom-built battery-operated ultrasound analog front end (UAFE). (a) The UAFE design includes a first stage power amplifier (PA), a passive Pi-pad attenuator circuit, and a second stage PA. (b) Simulation results for the second stage PA, conducted with circuit simulation software, demonstrated a gain of 59.6 dB at 500 kHz, with input and output voltages of 0.1 and 95 V, respectively. (c) All components including the first and second stage amplifiers and the passive Pi-pad attenuator circuit were assembled into a compact printed circuit board (PCB).

We designed a two-stage power amplifier (PA) to generate sufficient output voltage, enabling a single-element ultrasound transducer to achieve the necessary acoustic pressure for neuromodulation. This configuration utilizes a class A amplifier for high gain with linearity and incorporates a passive Pi-pad attenuator circuit to ensure impedance matching between the output of the first stage and the input of the second stage [Fig. 1(a)].^31^

The design of circuit schematic included a matching network (capacitor–inductor), a direct current block (capacitor), and a radio frequency choke (inductor).^32^ We achieved a gain of 41.6 dB and an output voltage of 12 V from the first stage amplifier with an input voltage of 0.1 V, and a gain of 17.9 dB and an output voltage of 95 V from the second stage amplifier with an input voltage of 12 V [Fig. 1(b)]. Based on these simulation results, we converted the schematic into a low-cost printed circuit board (PCB) for the UAFE with dimensions of 3.8 inches by 2.5 inches [Fig. 1(c) and supplementary material, Fig. S1].

### Performance evaluation of developed battery-powered UAFE

**FIG. 2.**
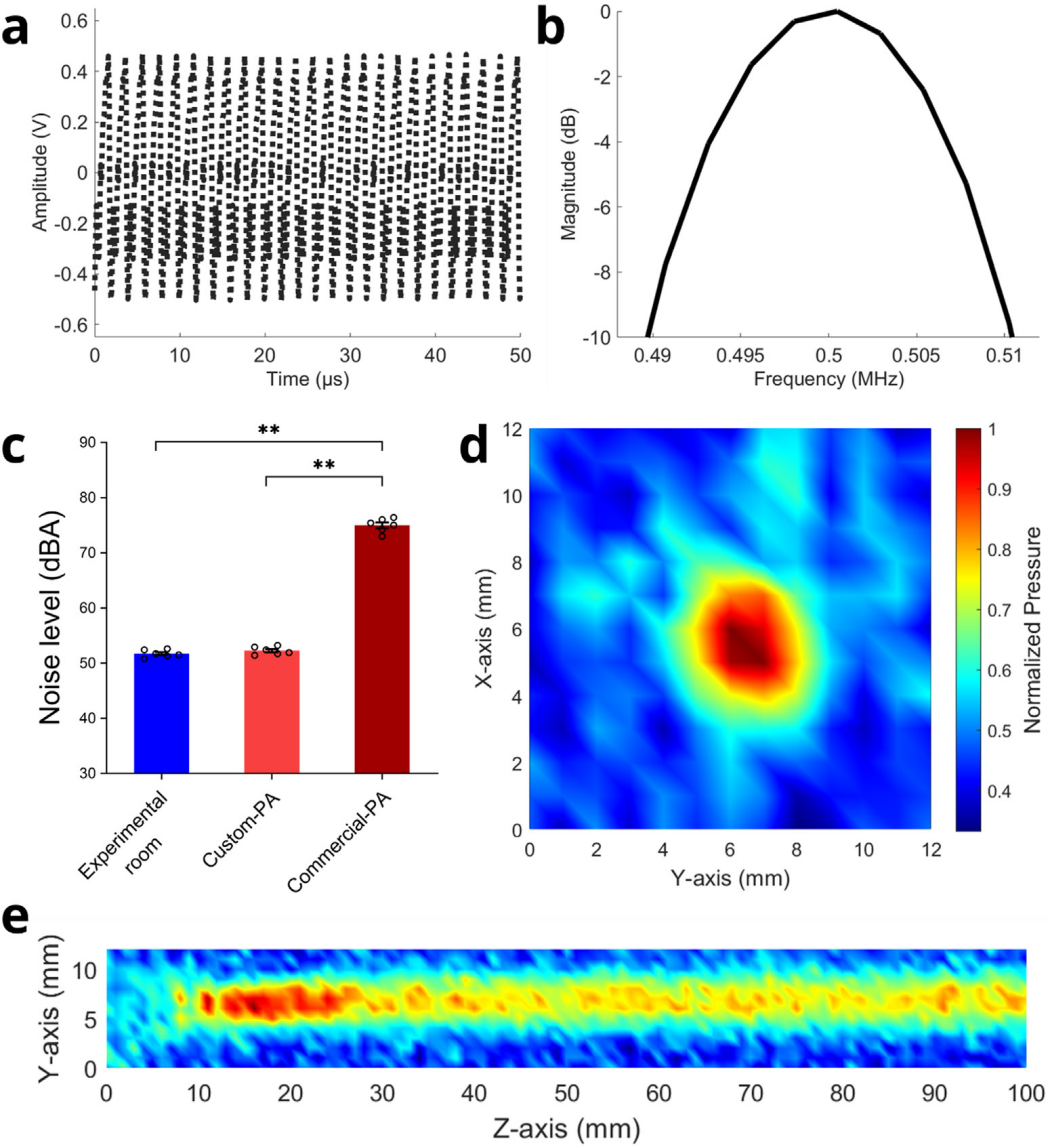
Measured performance of the custom-built UAFE. (a)–(b) The total gain of the two-stage PA was measured at 53.6 dB. The output waveform was recorded with a 40 dB attenuator to prevent damage to the oscilloscope. (c) Operating on battery power, the noise level of the custom-built UAFE was comparable to the ambient noise level of the experimental room, but significantly different from the noise level recorded during the operation of the commercial PA. (d)–(e) Normalized ultrasound beam profiles of the single-element transducer driven by the custom-built and battery-operated UAFE in free water.

We measured the maximum output waveform by applying external power supplies to the circuit board to maintain the maximum output voltage using an oscilloscope (DSO7104A, Keysight Technologies, Inc., Santa Rosa, CA, USA). Figure 2(a) illustrates that the gain and output voltage of the two-stage PA were measured to be 53.6 dB and 96 V, respectively, when the input voltage to the first stage amplifier was 0.2 V. These measurements are comparable with the simulation results (Table I). Furthermore, when comparing the custom-built UAFE with a commercial 50-dB power amplifier (2100L, Electronics & Innovation, Ltd., Rochester, NY, USA), the noise levels in A-weighted decibels (dBA) during operation of the battery-powered UAFE were significantly lower [^**^p = 0.0022 < 0.01, N = 6 trials, Fig. 2(b)] and comparable to the background noise levels of the experimental room [*p* > 0.05, N = 6 trials, Fig. 2(b)]. Additionally, the custom-built UAFE features a compact design and a cost-effective PCB board while achieving a high-amplitude output voltage over 50 V.

**TABLE I.** The simulated and measured total gain of the two-stage power amplifier (PA).

Target	Input (V)	Output (V)	Gain (dB)
Simulation	0.1	95	59.6
Measurement	0.2	96	53.6

Next, we quantified the performance of the custom-built battery-powered UAFE when integrated with a single-element transducer for neuromodulation based on prior studies.^33^ The peak-to-peak pressure of 148 kPa was measured from the single-element transducer operating at a center frequency of 500 kHz (AT31529, Blatek Industries, Inc., State College, PA, USA) in free water. This pressure level was selected to align with the targeted acoustic pressure used in previous studies.^23,34^ Figures 2(d) and 2(e) show the lateral and axial dimension of the acoustic focus driven by the custom-built and battery-operated UAFE, measured by using a needle hydrophone (HNR500, Onda Corp., Sunnyvale, CA, USA). The ultrasound focus obtained by applying external power supplies to the UAFE (i.e., doubling the output voltages of UAFE) had a lateral width of 5.7 mm, defined by the full width at half maximum (FWHM, −6 dB pressure) in a water tank (supplementary material, Fig. S2). The calibrated peak-to-peak pressure was 148 kPa when battery voltages of 9 and 18 V applied to the first and second stage PA, respectively, were used for UAFE in the animal pain experiments. In addition, the transducer operates at 91.5% efficiency compared to the commercial power amplifier, which was connected using a universal standard impedance for maximum power and voltage transfer. Specifically, this efficiency was determined by comparing the calibrated pressure and output voltage from both the developed UAFE and the commercial amplifier. This discrepancy may stem from an impedance mismatch, with the UAFE having an impedance of 15.2 Ω in relation to the transducer and its matching network. Additionally, the transducer's 85% efficiency when working with the UAFE was further calculated as the ratio of the transmitted acoustic power to the total input power by using a Coupler (CPL-LFLP, Electronics & Innovation, Ltd., Rochester, NY, USA).

### Characterization of tFUS targeting with acoustic simulation and behavioral testing setup

**FIG. 3.**
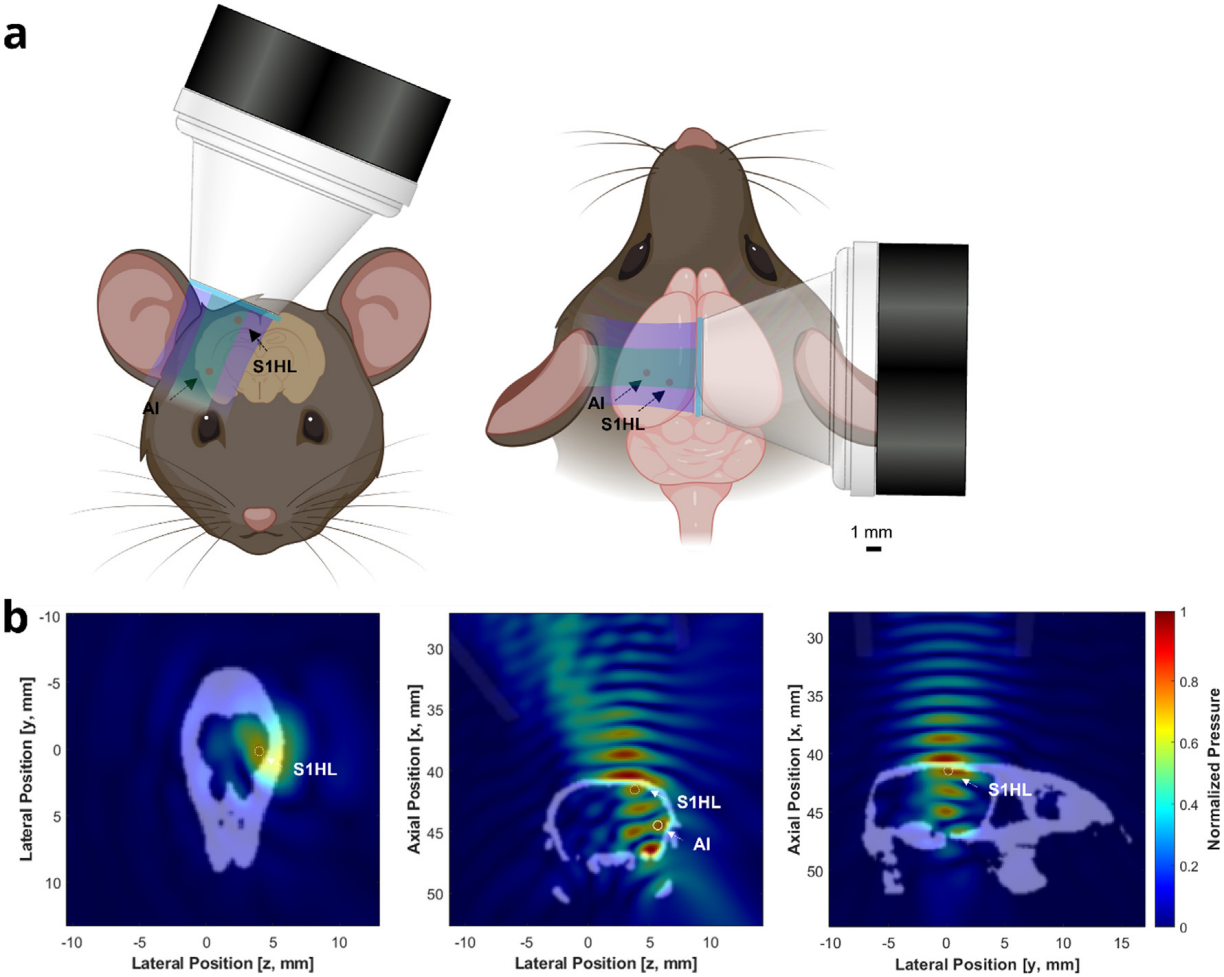
Schematic diagram illustrates the hypothesis of simultaneously targeting multiple pain processing brain circuits using a single-element transducer in a humanized sickle mouse model and simulation. (a) Targeting two primary pain processing circuits including the primary somatosensory cortex (S1HL) and agranular insular (AI) can significantly suppress heat-pain-related behaviors in aged sickle cell mice. Note that ultrasound beam shapes shown in green and purple represent the −3 and −6 dB pressure distributions relative to the spatial peak pressure, respectively. (b) The simulation results (left: transverse; middle: coronal; and right: sagittal sections) demonstrate that a single tFUS beam pathway can simultaneously target multiple pain processing brain circuits including S1HL and AI. Note that white represents cranial bones of mice and dark gray represents a collimator.

Given that specifically targeting S1HL or AI significantly suppressed pain-related behaviors in relatively younger BERK mice,^23^ we hypothesized that simultaneously targeting multiple pain-processing brain circuits including the S1HL and AI can induce significant amelioration of heat-pain-related behaviors in aged BERK mice [Fig. 3(a)]. To investigate the effectiveness of tFUS beam pathway using a single-element transducer, we performed ultrasound simulations using the k-wave acoustics toolbox for MATLAB.^35^ With an incidence angle of 23°, the pressure field of the single-element transducer connected with a collimator demonstrates that the proposed tFUS beam pathway can simultaneously target pain processing brain circuits including S1HL and AI [Fig. 3(b)], when compared to the pressure field of the multi-element transducer (supplementary material, Fig. S3). However, due to the inherent limitations of computational modeling,^36,37^ such as simplified tissue and transducer models and limited nonlinearity modeling, k-wave simulations were primarily utilized to estimate the ultrasound beam pathway and its specificity, rather than determining simulated pressure values. Consequently, for the precise pressure calculations, we conducted hydrophone measurements and found that measured pressure for S1HL and AI was 101–104 kPa in the presence of a mouse skull when using the single-element transducer with an incidence angle of 23°. On the other hand, the measured pressures at S1HL and AI were around 90 and 82 kPa, respectively, in the presence of the same mouse skull when using the multi-element transducer.^23^

**FIG. 4.**
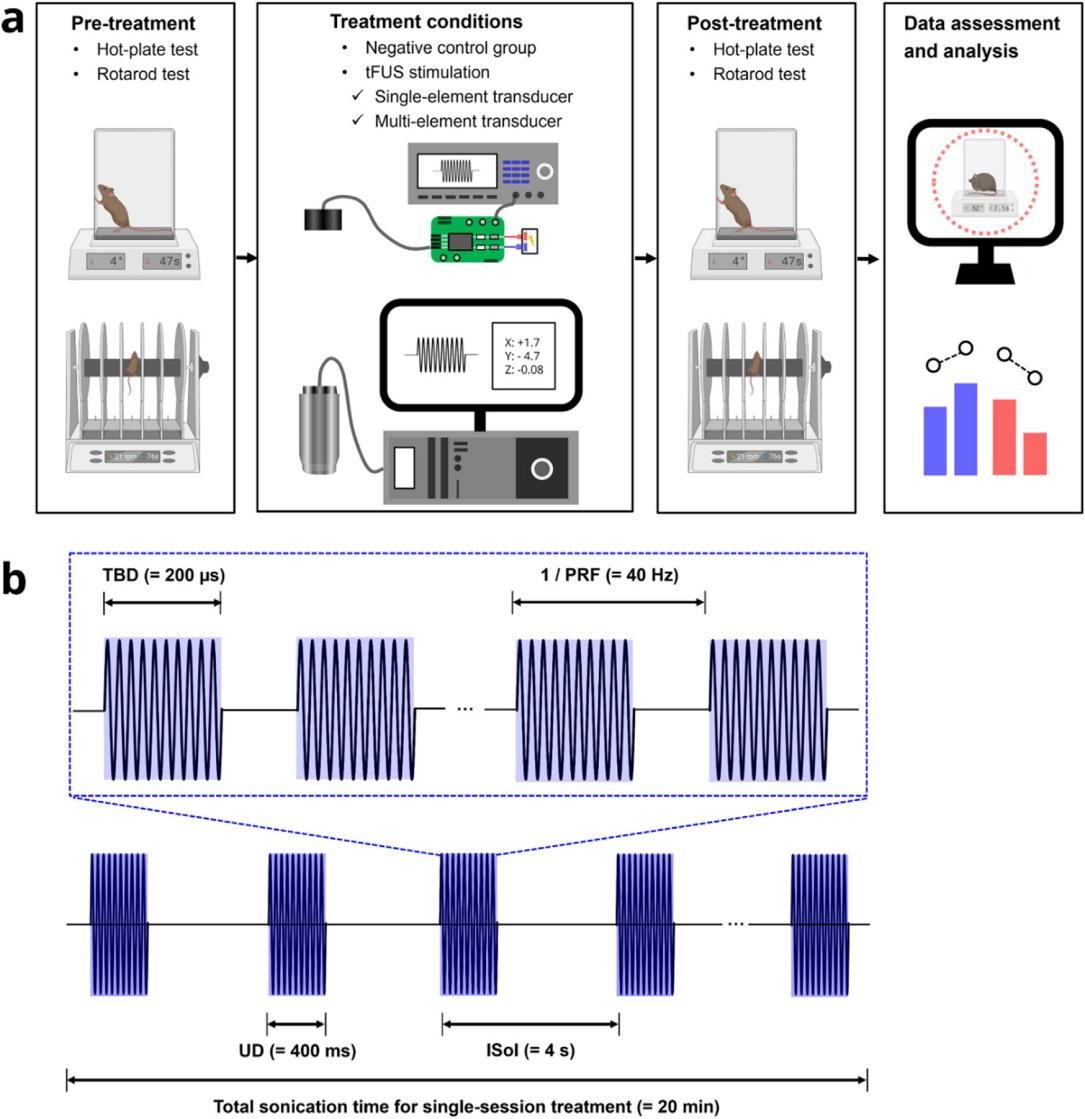
Behavioral testing setup and tFUS stimulation parameters. (a) The experimental setup comprised hot-plate and rotarod tests to assess pain-related behaviors and motor functions. tFUS stimulation was applied using a single-element transducer at a center frequency of 500 kHz for targeting multiple pain processing brain circuits simultaneously. For selective targeting of individual circuits, a 128-channel random array transducer was used. A negative control group, which followed all experimental procedures but did not receive tFUS stimulation, was included to rigorously evaluate the effect of tFUS on pain suppression. (b) The tFUS stimulation parameters included a single-session application at either 500 kHz or 1.5 MHz, with a tone burst duration (TBD) of 200 *μ*s, pulse repetition frequency (PRF) of 40 Hz, ultrasound duration (UD) of 400 ms, inter-sonication interval (ISoI) of 4 s, and a total sonication time of 20 min.

The behavioral experimental configuration included hot-plate and rotarod tests, tFUS stimulation with multiple stimulation control groups, and subsequent data assessment and analysis [Fig. 4(a)]. Detailed descriptions of the behavioral tests are provided in the Methods section. To evaluate the effects of tFUS stimulation in *in vivo* small animals, hot-plate and rotarod tests were conducted to assess heat pain-like behaviors and motor coordination separately before and after the treatment. To target multiple pain processing brain circuits including S1HL and AI, we used a single-element transducer operating at a center frequency of 500 kHz, excited by the battery-powered compact UAFE using the same volts in the performance evaluation step. For comparisons, we employed a 128-channel random array transducer (H276, Sonic Concepts, Inc., Bothell, WA, USA) to selectively target each pain processing brain circuit.^23^ Additionally, a negative control group was included in which the same experimental procedures were followed but without activating the ultrasound transducer. These controls allowed for a rigorous examination of the tFUS suppression effects. Brain structures including S1HL and AI were targeted by single-session tFUS with two different setups: a single-element ultrasound transducer at a center frequency of 500 kHz, a diameter of 25.4 mm, and a focal depth of 38.1 mm, and a 128-element ultrasound array transducer at 1.5 MHz, an aperture outer diameter of 15 mm, a radius of curvature of 8.5 mm, a focal depth of 2.78 mm. The parameters^23^ for both setups, which previously demonstrated on significant suppression of pain-related behaviors, selected and used a tone burst duration (TBD) of 200 *μ*s, a pulse repetition frequency (PRF) of 40 Hz, an ultrasound duration (UD) of 400 ms, an inter-sonication interval (ISoI) of 4 s, and a total sonication time of 20 min [Fig. 4(b)].

### Effect of age and tFUS stimulation on the heat hyperalgesia and motor coordination in aged BERK mice

**FIG. 5.**
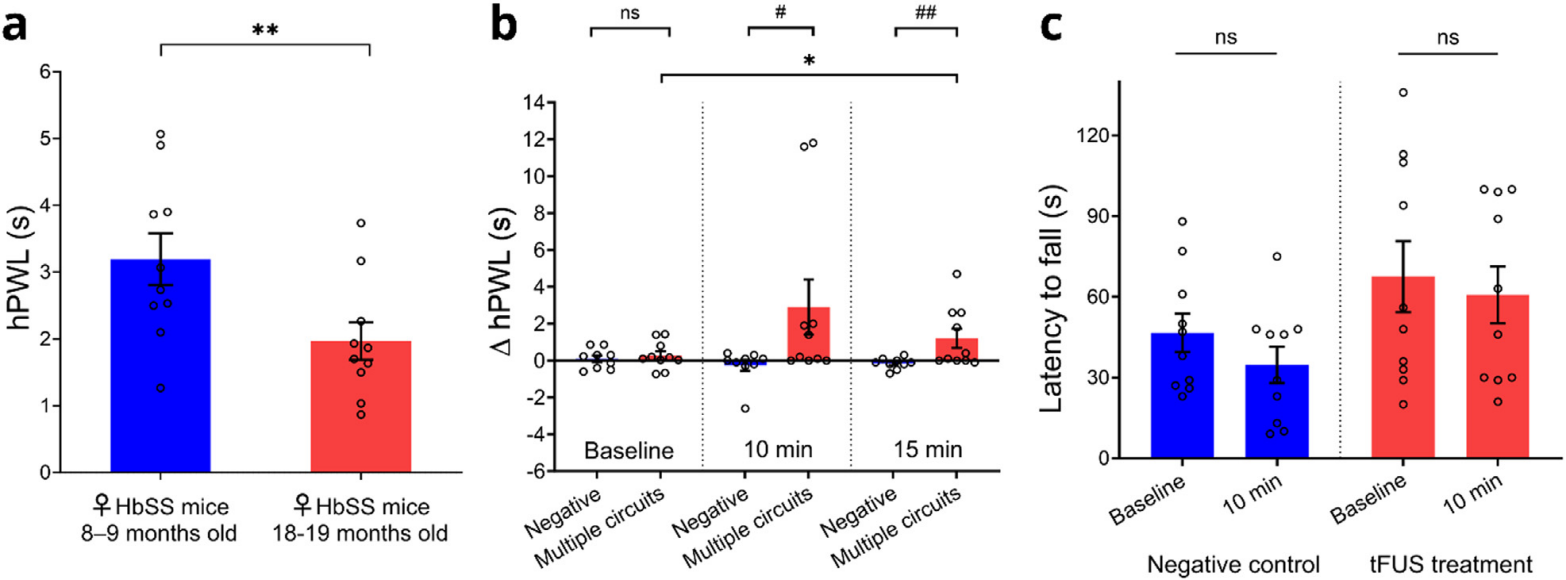
Animal behavioral study with tFUS stimulation. (a) A significantly higher heat hyperalgesia, indicated by a lower hind paw withdrawal latency (hPWL), was observed in older female HbSS-BERK mice (1.97 ± 0.26; N = 10; 18–19 months old) compared to younger female mice (3.19 ± 0.40; N = 10; 8–9 months old). (b) After single-session tFUS stimulation with a pulse repetition frequency (PRF) of 40 Hz targeting multiple pain processing circuits using a single-element transducer and a battery-operated UAFE, the averaged Δ hPWL values (2.90 ± 1.49 for 10 min and 1.21 ± 0.52 for 15 min; N = 10) were significantly increased compared to both baseline values (0.28 ± 0.24; N = 10) and the negative control group (−0.26 ± 0.30 for 10 min and −0.16 ± 0.10 for 15 min; N = 9). (c) The average time spent on the rotarod did not significantly differ from baseline values (46.60 ± 7.15 for negative control and 67.5 ± 13.20 for tFUS treatment; N = 5) in both the negative control (34.70 ± 6.79; N = 5) and a single-session treatment group (60.70 ± 10.56; N = 5) with the single-element transducer, indicating no significant adverse effect on motor coordination and balance. ns: not significant, ^*^p < 0.05, ^*^*p < 0.01 using t-test with Wilcoxon signed rank test; ^#^*p* < 0.05, ^##^*p* < 0.01 using t-test with Mann–Whitney test.

Given the challenges of pain treatment in older adults,^23,38,39^ particularly females who have been shown to experience more severe pain than male counterparts,^27,28^ we examined age-related behavioral responses to noxious heat stimuli by comparing hind paw withdrawal latency (hPWL) values between relatively younger (8-to-9-month-old) and older (18-to-19-month-old) female humanized Berkley sickle cell mice (HbSS-BERK). We observed a significant decrease in hPWL in older BERK mice compared to younger mice [^**^p = 0.0039 < 0.01, N = 10 mice, Fig. 5(a)] suggestive of increased pain hypersensitivity. This finding is consistent with prior studies showing that older subjects exhibit significantly higher heat hyperalgesia than younger ones.^23,29^ Based on the observed significantly higher heat hyperalgesia in older female BERK mice, we investigated whether simultaneously targeting pain processing brain circuits could result in a significant suppression of heat hyperalgesia using tFUS. Applying tFUS pulses with a PRF of 40 Hz to target multiple brain circuits including the left S1HL and AI using a single-element transducer and battery-powered UAFE led to a notable increase in the average Δ hPWL for 10 and 15 min after tFUS stimulation compared to the negative control [^*^p = 0.0284 < 0.05, N = 9 mice for 10 min; ^**^p = 0.0065 < 0.01, N = 9 mice for 15 min, Fig. 5(b)]. This result indicates a significant amelioration of heat hyperalgesia. We then investigated whether targeting a single pain processing brain circuit could also suppress pain-related behaviors. For this test, we applied a single-session tFUS stimulation using a 128-element random array transducer to the S1HL or AI in older female BERK mice while maintaining the same behavioral procedures. We observed that the Δ hPWL after targeting a single brain circuit including S1HL and AI with the 128-element random array transducer was not significantly different from the negative control (*p* > 0.05, N = 7–8 mice for 10 and 15 min, supplementary material, Fig. S4). Collectively, these results support that single-session tFUS stimulation with a PRF of 40 Hz targeting multiple pain processing brain circuits appears to be statistically significant beneficial effects for ameliorating heat hyperalgesia in aged female BERK mice.

We next examined the impact of simultaneously targeting pain processing brain circuits on motor coordination and balance using the rotarod test to assess the safety of our newly developed device. The rotarod test is a well-established method for evaluating motor coordination and balance by measuring the time spent on a rotating rod.^40,41^ After single-session tFUS stimulation with a PRF of 40 Hz, no significant difference in the average time spent on the rotarod was observed compared to the pre-stimulation baseline [*p* > 0.05, N = 5 mice, Fig. 5(c)]. Similarly, the latency to fall on the rotarod for the negative control group was not significantly different from baseline values [*p* > 0.05, N = 5 mice, Fig. 5(c)]. These results suggest that single-session tFUS stimulation targeting multiple brain structures does not induce significant adverse effects on motor function in aged female BERK mice.

## DISCUSSION

We have developed a compact, low-cost, and battery-powered UAFE with a novel circuit design and show a significant amelioration of heat hyperalgesia in aged female BERK mice through simultaneous modulation of multiple pain-processing circuits without affecting significant adverse effects on the motor function. The present results suggest a potential solution for home-based pain treatment for senior subjects who experience severe pain at increased risk for adverse drug reactions.

Currently, chronic pain, linked to numerous physical and mental conditions, is emerging as a critical health concern driven by following factors. First, chronic pain tends to worsen over time and becomes increasingly difficult to treat due to nociceptive plasticity.^42^ Moreover, persistent pain, coupled with an increased likelihood of experiencing unwanted drug side effects, becomes more pronounced with age.^7,43^ Thus, well-established pain management strategies for older adults are urgently needed. Additionally, with its unique features compared to other noninvasive brain stimulation methods, tFUS stimulation has demonstrated feasibility for managing pain by targeting specific brain circuits in prior studies.^21–25^ For example, precise sonication to the right anterior thalamus using a single-element transducer at the fundamental frequency of 650 kHz led to a suppression of thermal pain sensitivity in 11 healthy individuals with a mean age of 24.5.^21^ Targeting posterior insula using a single-element transducer at the fundamental frequency of 500 kHz attenuated amplitude of the heartbeat evoked potential in 16 healthy human participants with a mean age of 25.7.^22^ Specifically stimulating the S1HL or insula resulted in a notable amelioration of pain-related behaviors in relatively younger sickle mice ranging from 5 to 9 month-old.^23^ In comparison with studies conducted on younger subjects, a few investigations demonstrated an improvement in subjective mood in 31 chronic pain patients with a mean age of 52.8 by applying ultrasound energy at the posterior frontal cortex using a linear array at the center frequency of 8 MHz^24^ and showed a reduction of pain in 23 patients with chronic pain by targeting the anterior cingulate cortex at the center frequency of 0.65 MHz.^25^ However, those studies primarily involved middle-aged adults with a mean age of 42.5 years^24^ as well as chronic pain patients with an average age of 46.6 years.^25^ Therefore, we sought to develop innovative pain treatment strategies for older populations by investigating the effect of a new tFUS stimulation paradigm in the humanized sickle mouse model of chronic hyperalgesia, one of the most challenging pain phenotypes. To quantify nociception in animal pain studies, we provided a quantitative metric and conducted a statistical analysis to evaluate the effect of tFUS on heat pain hypersensitivity, which is consistent with prior studies.^23,29,30^ We found a statistically significant reduction of heat hyperalgesia, which is represented as a remarkable increase in Δ hPWL of 2.90 ± 1.49 for 10 min and 1.21 ± 0.52 for 15 min (N = 10), in aged female BERK mice with 18–21 months old (corresponding to humans ranging from 56 to 69 years old^44^) by simultaneously targeting multiple pain processing circuits including S1HL and AI when compared with its baseline value of 0.28 ± 0.24 (N = 10) and values from the negative control group of −0.26 ± 0.30 for 10 min and −0.16 ± 0.10 for 15 min (N = 9). The experimental evidence has paved the way for new pain management strategies for older subjects to fulfill the unmet need.

Given that^45^ older adults with chronic pain often struggle with impaired mobility, limited financial resources, and find it difficult to make daily trips to the medical facility, our low-cost and compact UAFE device for driving ultrasound transducer is a first step toward the development of potentially in-home usable pain treatment by addressing the challenges faced by the increasing population of the elderly in our society. Importantly, battery-powered UAFE was designed to generate sufficiently high-amplitude output voltages without disturbing human hearing^46^ and then developed into a compact and low-cost PCB board with 3.8 inches by 2.5 inches. This is in remarkable contrast to a commercially available 50-dB transmitter, which has dimensions of 18.4 inches by 16.5 inches, high fabrication costs, and high noise levels. In addition, such a developed device should be equipped with additional key features including noninvasive treatment, high tolerance, and user-friendly technology, which aligns with the advantages of using therapeutic ultrasound.^47^ Building on these notable features, a low peak acoustic pressure level (≤150 kPa in free water) emitted by the battery-operated UAFE was used to test our hypothesis in animal behavioral experiments. Regarding safety concerns and the potential for clinical translation, the current acoustic pressure level used in our study is significantly lower than the levels reported in the Food and Drug Administration (FDA) standard for diagnostic ultrasound imaging^48^ and prior studies.^49,50^ However, it will need to be scaled up for human applications, considering the thicker skull and larger brain volumes compared to small animals. Consequently, the input voltage for the UAFE will need to be increased accordingly, and at this pressure level, comprehensive investigations into the UAFE performance as well as structural integrity of tissues and nuclear morphology in mice and rats will be performed to support the further development of in-home usable pain treatment.

The findings of our study are promising, but several questions and limitations should be considered. First, gender bias needs to be determined considering gender-related differences in biological and psychosocial factors individually. Since majority (about 72%) of chronic pain patients are females exhibiting greater pain sensitivity than age-matched males,^51,52^ we first evaluated the efficacy of tFUS in older female subjects to address high-priority research needs and found a significant reduction of heat hyperalgesia in aged female BERK mice. However, further investigation is warranted to better understand pain and tFUS-based pain management on aged men and women as well as to explore its effects on different types of pain stimuli such as cold and mechanical hyperalgesia. Second, we provided evidence-based suppression of heat hyperalgesia by investigating the effects of tFUS on multiple pain-processing circuits, including the S1HL and AI. However, as tFUS stimulation also covers motor cortex, a further investigation of how tFUS interacts with motor-related brain regions is needed given the analgesic effects of TMS and tDCS stimulation to the motor cortex.^53,54^ Thus, we further explored whether heat pain-related behaviors can be significantly changed by a precise primary motor cortex (M1) stimulation using 128-element random array transducer. Similar to other brain regions, single-session tFUS stimulation of the M1 did not induce a significant change in Δ hPWL compared to the negative control (*p* > 0.05, N = 7 mice for 10 and 15 min, supplementary material, Fig. S4). Based on the experimental results, targeting motor-related brain regions did not lead to a significant modulatory effect on heat hyperalgesia in aged female BERK mice. Given the results, additional studies are needed to implement multiple target beamforming scenarios with a 128-element random array transducer. This enables us to selectively target specific pain-processing brain circuits to enhance the modulatory effect while minimizing the confounding impact of targeting unwanted brain regions. Specifically, prior studies demonstrated that certain brain regions predominantly involved in pain processing^55,56^ include S1, the secondary somatosensory cortex (S2), insula, and anterior cingulate cortex (ACC) along with current stimulation paradigm. Thus, further studies are needed to investigate the potential effects of stimulating other pain-processing brain circuits, such as S2, located between primary somatosensory cortex (S1), and AI. Third, previous neuroimaging studies have shown that the S1 and insula are key brain locations involved in pain perception and modulation.^56,57^ Additionally, a recent study has demonstrated a significant suppressive effect of tFUS on the pain-related behaviors in relatively younger BERK mice by targeting each brain location of S1HL and AI.^23^ Here, our findings along with the hypothesis suggest that simultaneously targeting multiple pain-processing circuits including S1HL and AI is effective in reducing heat hyperalgesia in aged female BERK mice. The hypothesis was further indirectly validated by comparing the effects of single-target stimulation in the same mice (supplementary material, Fig. S4). Based on these results, having capability to target different brain circuits could offer greater benefits than targeting a single location. Moreover, the size of the brain regions within S1HL or AI being treated may influence the suppression of pain-related behaviors given that the single-element transducer may be better suited for covering larger areas (Fig. 3), while the multi-element transducer offers advantages in targeting narrower, more specific brain regions (supplementary material, Fig. S3). Although the detailed biological mechanism remains to be elucidated, the results presented here indicate that the inhibitory effects of tFUS stimulation with a PRF of 40 Hz at multiple pain-processing brain circuits including the S1HL and AI may accumulate and be possibly amplified. This accumulation and amplification could potentially lead to a greater modulatory effect on the pain descending pathway [Fig. 5(b)], which was supported by a prior study^58^ demonstrating the effectiveness of combined electrical stimulation of the periaqueductal gray matter (PAG) and sensory thalamus in 11 patients. Collectively, this new stimulation paradigm is promising; however, it may not be effective for all subjects as observed in our study [Fig. 5(b)] and the reported study,^58^ considering the heterogeneity in pain between different individuals and under variable primary pathological condition. Therefore, optimization and personalization of the stimulation strategy are necessary to maximize the modulatory effect for various older subjects. This will involve adjusting parameters and selecting the appropriate number and types of pain-processing brain circuits. Finally, the current UAFE design includes a two-stage amplifier with a Pi-pad attenuator circuit, demonstrating translational potential with its compact and low-cost features, especially when replacing the commercial PA. However, additional strategies are needed to develop improved impedance matching network^59^ as well as a standalone portable tFUS treatment device including a field-programmable gate array for user interfaces to adjust stimulation dosage and a wearable ultrasound transducer. Once we establish a multi-region brain stimulation strategy and a standalone pocket-sized device with the impedance matching network, these advancements could become a significant stepping-stone toward the entirely home-based self-administration for chronic pain treatment in the elderly.

## CONCLUSIONS

A pain treatment strategy for senior subjects using noninvasive tFUS is demonstrated. We found that simultaneously targeting multiple pain-processing circuits effectively suppresses heat pain-related behaviors in aged female sickle mice without adverse effects on motor coordination and balance. Additionally, brain stimulation was achieved using a battery-powered, compact, and low-cost UAFE that generates a sufficiently high-amplitude output voltage with significantly low noise levels. The experimental results suggest that this approach can provide effective and noninvasive pain management as an alternative to opioids, paving the way for advancements in home-based and self-administered tFUS treatment for older adults suffering from chronic pain and painful conditions.

## METHODS

### Simulation of circuit block diagram for the designed custom-built UAFE

The first stage amplifier, which utilizes a bipolar transistor (BFG35, NXP Semiconductors, Eindhoven, Netherlands), was initially designed and simulated using circuit simulation software (Advanced Design System, Keysight, Santa Rosa, CA, USA) [Fig. 1(a)]. Following this, the second stage amplifier, employing a power metal-oxide-semiconductor field-effect transistor (PD57018, STMicroelectronics, Geneva, Switzerland), was designed to further increase gain [Fig. 1(a)]. Based on the simulation results, the schematic for the two-stage amplifier was converted into a PCB. Electrical components were soldered onto the PCB, and the component values of the first and second stage amplifiers along with the passive Pi-pad attenuator were meticulously tuned to achieve optimal performance^59,60^ [Fig. 1(c)].

### Performance evaluation of the custom-built UAFE

Noise levels in A-weighted decibels (dBA) were measured using a digital sound level meter (HT-80A, Risepro) during the operation of the custom-built UAFE with batteries, and a commercial 50-dB power amplifier (2100L, Electronics & Innovation, Ltd., Rochester, NY, USA), in the experimental room. Each measurement was repeated six times with an inter-measurement interval of 30 seconds and then averaged.

To calibrate the output pressure and field of the single-element transducer driven by the custom-built UAFE,^33,61^ we used a three-dimensional scanning system connected with a three-axis motorized positioning stage (XSlide, Velmex, Inc., Bloomfield, NY, USA) and a needle hydrophone (HNR500, Onda Corp., Sunnyvale, CA, USA). With a step size of 1 mm, the input voltages to the transducer at various locations were converted to ultrasound pressure values using the pre-determined calibration factor provided by the manufacturer. To smooth the colors between grid points, the MATLAB function *interp* was used.

### Animal

Humanized transgenic female Berkley sickle cell mice (HbSS-BERK) were used for the study. These mice were on a mixed genetic background (FVB/N, 129, DBA/2, C57BL/6, and Black Swiss) with murine α and β globin knockouts (α^−/−^ and β^−/−^) expressing >99% human sickle hemoglobin S (HbS). The BERK mice demonstrate several features of sickle cell disease including chronic pain.^62,63^ Age-matched female BERK mice (8–9 and 18–19 month-old) were used to compare age-related behavior changes in response to heat pain. Female BERK mice aged 18–21 months were used to investigate the efficacy of tFUS stimulation on the pain-associated behaviors.

During the tFUS treatment, mouse was anesthetized using 3% isoflurane with a 1 L/min oxygen flow for induction and 1%–1.25% isoflurane with a 0.2–0.3 L/min oxygen flow for maintenance. To maintain body temperature and protect the eyes, a heating pad and ophthalmic ointment were used, respectively. The rectal temperature was frequently measured to check mice's core body temperature during the experiments. The study was approved by the Institutional Animal Care and Use Committee of Carnegie Mellon University (PROTO201900017) complied with National Institutes of Health guidelines.

### Evaluation of tFUS targeting with acoustic field simulation and characterization of behavioral testing setup

To estimate the targeted brain circuits using ultrasound transducers, including a single-element transducer and a 128-element random array transducer, we utilized the Allen Mouse Brain Atlas. Targeting procedures for the 128-element random array transducer were followed as described previously.^23^ For precise placement of the single-element ultrasound transducer on the brain, the transducer was initially positioned at the intersection between the posterior end of the zygomatic structure and the mouse's rostral-caudal line and then moved posteriorly by a distance of 3.5 mm. A collimator connected to the transducer was positioned at an incidence angle of 23° to simultaneously stimulate multiple pain processing circuits including S1HL and AI. The spatial profiles of the ultrasound beam were further studied by k-wave acoustics toolbox for MATLAB^35^ with a meshed mouse skull model. The mouse bone model was generated by InVesalius (version 3.1.1, Renato Archer Information Technology Center, Brazil) from its full body CT images, and segmented and meshed in SOLIDWORKS (version 2023, Dassault Systèmes SE, France) for reducing computational requirements in subsequent simulation procedures (supplementary material, Fig. S5). In all simulations, there were mainly four sets of parameters including kgrid, medium, source, and sensor. First, kgrid was defined in 3D space with a dimension of [x,y,z] = [70,70,70] mm, a grid spacing of dx = 0.15 mm, and a Courant-Friedrichs-Lewy (CFL) number of 0.05 to ensure the stability of simulations. A series of continuous wave (CW) signals were generated as the source. Source frequency ( $f_{0}$) was set to 500 kHz according to the experimental setup, and source pressure was set to 25.82 kPa to match the free-field peak-pressure measurements. Moreover, the sensor covered the all-grid points in the 3D space to record acoustic pressure during simulations. In addition, medium parameters were assigned with density ( ρ) in water, collimator, and skull of 1000, 1160, and 2200 kg/m^3^; speed of sound (c) in water, collimator, and skull of 1500, 2410, and 3100 m/s; and power law absorption coefficient ( α) in water, collimator, and skull of 3.02 × 10^−5^, 9.54, and 12.16 dB/MHz/cm based on the prior study,^34,64,65^

$$\psi =1-\frac{H}{1000},$$ (1)

$$\alpha_{\text{skull}}=\alpha_{\text{min},\text{skull}}+\left(\alpha_{\text{max},\text{skull}}-\alpha_{\text{min},\text{skull}}\right)\psi^{0.5}.$$ (2)

Specifically, the α value of the skull model ( $\alpha_{\text{skull}}$) was calculated through Eq. (1) and (2), in which $\alpha_{\text{min},\text{skull}}=21.5$ and $\alpha_{\text{max},\text{skull}}=208.9$ (Np MHz^−1^ m^−1^) were determined.^53^ In addition, a single value H=600 (Hounsfield units) was chosen based on a mean HU value of 596.80, obtained from the raw CT image using a threshold range of 400–3000 HU. This approach was informed by the literature, where HU values for mouse bone typically exceed 200 HU,^66^ and it was used to simplify the complexity of modeling and to determine the porosity ( ψ). The source parameters including Radius of Curvature (ROC) and source pressure were calibrated to match its real-world performance based on the hydrophone data obtained in a water tank. Furthermore, spatial locations of the skull and collimator models were placed based on the experimental setup.

Behavioral experiments using hot-plate and rotarod tests were performed during the light cycle for mice. Prior to testing, all BERK mice were acclimated to the experimental environment for 30 min and to each behavioral test apparatus for at least 2 days: 30 min for the hot-plate test and 10 min for the rotarod test. In the hot-plate test, the temperature of the hot-plate (Ugo Basile, Collegeville, PA, USA) was set to 52 °C for the BERK mice based on previously conducted temperature calibration.^23^ On the day of measurement, following the habituation period, mice were gently placed onto the surface of the hot-plate. The hind paw withdrawal latency (hPWL) was recorded and calculated as the time taken for the mouse to exhibit any nociceptive response such as hind paw licking, flicking, or jumping off the hot-plate. If no response was observed within 20 s, the mouse was promptly removed from the hot-plate to prevent potential tissue injury. The baseline measurement was conducted in triplicate with a 5-min inter-measurement interval, and the average value was calculated. After treatment with either tFUS or negative control, the reaction time to painful heat stimuli was assessed at 10 and 15 min following a single 20-min session of treatment. All hot-plate tests were video-recorded to conduct a blind assessment. In the rotarod test, motor coordination and balance were evaluated using an accelerating rotarod apparatus^40,41^ (Ugo Basile, Collegeville, PA, USA). On the day of measurement, following habituation, mice were gently placed onto the rotating rod, which accelerated from three revolutions per minute (rpm) to 72 rpm over a 300-s period. After falling up to three times, the mouse was immediately placed back onto the rotating rod. The latency to fall was recorded and calculated as the time it took for the mouse to fall off the rod. Following treatment with either tFUS or the negative control, the response time on the rotarod was measured 10 min after a single 20-min session. All rotarod tests were video-recorded for a subsequent analysis. To control for potential confounding variables^67,68^ such as learning effects or habituation, measurements were conducted with an inter-experimental interval of 6–7 days.^23^ Given the ultrasound focus on targeting the left hemisphere of the brain using ultrasound transducers including both the single-element and 128-element random array transducers, the unilateral stimulation effect was assessed. This was calculated as the difference between contralateral and ipsilateral hind paw withdrawal latency (Δ hPWL), as defined by the following formula:^23,69^ Δ hPWL (s) = contralateral hPWL - ipsilateral hPWL.

### Statistical analysis

The results were presented as mean ± standard error of the mean (s.e.m.). Statistical analysis was conducted using commercial software (GraphPad Prism, GraphPad Software, San Diego, CA, USA). Non-parametric t-test with Wilcoxon matched-pairs signed rank test was used to compare behavioral responses to the pre-stimulation baseline. Statistical significance was defined as follows: ns: not significant; ^*^p < 0.05, ^**^p < 0.01. In addition, non-parametric t-test with Mann–Whitney test was used to compare behavioral responses to values from other treatment conditions within the same measurement time. Statistical significance was defined as follows: ns: not significant; ^#^*p* < 0.05, ^##^*p* < 0.01.

## SUPPLEMENTARY MATERIAL

See the supplementary material for additional details and results along with Figs. S1–S5.

## Data Availability

The data that support the findings of this study are available within the article and its supplementary material. Additional data can be found in Figshare repository at: Behavior assessments and analyses: https://doi.org/10.6084/m9.figshare.28053902; Ultrasound Characterizations: Ex-vivo Scanning: https://doi.org/10.6084/m9.figshare.28054031.
